# Homoisoflavonoids and Chalcones Isolated from *Haematoxylum campechianum* L., with Spasmolytic Activity

**DOI:** 10.3390/molecules22091405

**Published:** 2017-08-24

**Authors:** Armando Escobar-Ramos, Carlos Ernesto Lobato-García, Alejandro Zamilpa, Abraham Gómez-Rivera, Jaime Tortoriello, Manasés González-Cortazar

**Affiliations:** 1 Academics Division of Basic Sciences, University Juárez Autonomous of Tabasco, Highway Cunduacán-Jalpa Km. 0.5, Cunduacán Tabasco 86690, Mexico; armando.escobar@ujat.mx (A.E.-R.); abgori@gmail.com (A.G.-R.); 2 Southern Biomedical Research, Mexican Institute of Social Security, Argentina No. 1, Col. Centro, Xochitepec, 62790, Mexico; azamilpa_2000@yahoo.com.mx (A.Z.); jtortora2@yahoo.es (J.T.)

**Keywords:** homoisoflavonoids, *Haematoxylum campechianum*, chalcone, spasmolytic

## Abstract

*Haematoxylum campechianum* is a medicinal plant employed as an astringent to purify the blood and to treat stomach problems such as diarrhea and dysentery. A bio-guided chemical fractionation of the methanolic extract obtained from this plant allowed for the isolation of five compounds: two chalcones known as sappanchalcone (**1**); 3-deoxysappanchalcone (**2**); three homoisoflavonoids known as hematoxylol A (**3**); 4-*O*-methylhematoxylol (**4**); and, hematoxin (**5**). The spasmolytic activity was determined in an in vitro model (electrically induced contractions of guinea pig ileum), and allowed to demonstrate that the methanolic extract (EC_50_ = 62.11 ± 3.23) fractions HcF7 (EC_50_ = 61.75 ± 3.55) and HcF9 (EC_50_ = 125.5 ± 10.65) and compounds **1** (EC_50_ = 16.06 ± 2.15) and **2** (EC_50_ = 25.37 ± 3.47) of *Haematoxylum campechianum* present significant relaxing activity as compared to papaverine (EC_50_ = 20.08 ± 2.0) as a positive control.

## 1. Introduction

The Haematoxilon genus (Leguminosae) has two species: *Haematoxylum campechianum* and *Haematoxylum brassileto*. Both are native trees of Central America and belong to the family of the Fabaceae and the subfamily Caesalpinioideae [1]. *H. campechianum* in Mexico is known as the “red wood”, “black wood”, and “Campeche wood” because it originally was found on the shores of the Gulf of Mexico, in the state of Campeche [2]. The heartwood of this tree is an important source for the well-known dye hematoxylin which is a staining agent employed in histology [3,4,5]; also, the extract from the wood has been used as a sweetener [6]. Traditional medicine refers to the use of the heartwood of this plant for the treatment of depression, kidney disorders, heart problems, toothache, fever, diarrhea, and hemorrhoids [7,8]; and the branches are mainly used to remove toxins [9]. Chemical studies of this plant lead to identifying: hematoxylin, which by oxidation produces hematein [6]; brazilin [10,11], which was also isolated from *Caesalpinia echinata* (Leguminosae); hematoxylol A (**3**) [6]; protossapanin A and B; sappanchalcona (**1**); 3-deoxisappanchalcone (**2**), isolated from *Caesalpinia sappan* L., tetra-*O*-methylhematoxylol B [6,12]; and, trimethylether of protosappanin B [6], as well as: hematoxylol, epihematoxylol, 4-*O*-methylhematoxylol, 4-*O*-methylepihematoxylol, sappanene, hematoxylene, hematoxylone, hematoxin, epihematoxin, hematoxilol B, and isohematoxylin. It is noteworthy that these compounds showed activity on the inhibition of tyrosine kinase, being hematoxylin, hematoxylol A (**3**) and epihematoxylol B the most active compounds [13,14,15]. The flavonoid sapanchalcone which also was isolated from *Caesalpinia sappan* L., has an anti-inflammatory effect in a model of rheumatoid arthritis in mice [16]. However, the biological studies reported so far have only been focused on isolated compounds, but not on extracts and fractions for the heartwood of *H. campechianum*. Our interest is focused on compounds of the flavonoid type because they have been shown to have important biological activity on disorders of the central nervous system [17,18]. The aim of this study was to demonstrate the spasmolytic activity of the methanolic extract. Five fractions (HcF5-HcF9) obtained from the chromatographic separation of this extract exhibited five isolated compounds: two chalcones (compounds **1** and **2**) and three homoisoflavonoids structurally derived from hematoxylin (compounds **3**, **4** and **5**), all of them have been reported previously for this plant [6,11]. The biological model employed was the in vitro electrical stimulation of the isolated ileum of guinea pig. Additionally, all five compounds were characterized by one and two-dimensional NMR.

## 2. Results and Discussion

### 2.1. Evaluation of the Ethanolic Extract and Fractions

**Figure 1:**
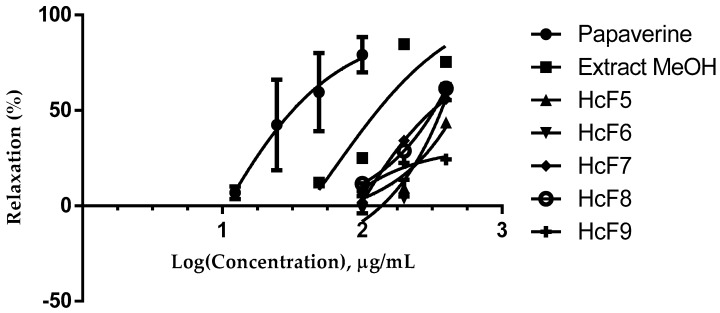
Concentration-response curves of the methanolic extract, HcF5 to HcF9 fractions of *Haematoxilum campechianum* induced contractions in isolated guinea pig ileum compared with papaverine as control (+).

The results presented in Figure 1 demonstrate that the methanolic extract and the fractions of *H. campechianum* induced a concentration-dependent inhibition of the electrical stimulation induced contractions in guinea pig ileum, using papaverine as a positive control. The values of EC_50_ and E_max_ are presented in Table 1.

The methanol extract obtained by the maceration of the heartwood of *H. campechianum* showed inhibition of the electrically induced smooth muscle contraction (EC_50_ = 62.11 ± 3.23), which is similar to the HcF7 fraction (EC_50_ = 61.75 ± 3.55) obtained from the chromatographic separation; whereas, the HcF9 fraction (EC_50_ = 125.5 ± 10.65) presented the lower activity when compared with papaverine (EC_50_ = 20.08 ± 2.0). It is noteworthy that both the methanolic extract and the HcF7 fraction showed a similar effect to the one exerted by papaverine, as shown by the comparison of the Emax (85.16 ± 6.34 and 84.6 ± 1.7 versus 85.72 ± 2.8). The HcF5, HcF6, and HcF8 fractions showed no significant spasmolytic activity.

### 2.2. Evaluation of Compounds ***1***, ***2*** and ***3a**–**5a***

**Figure 2:**
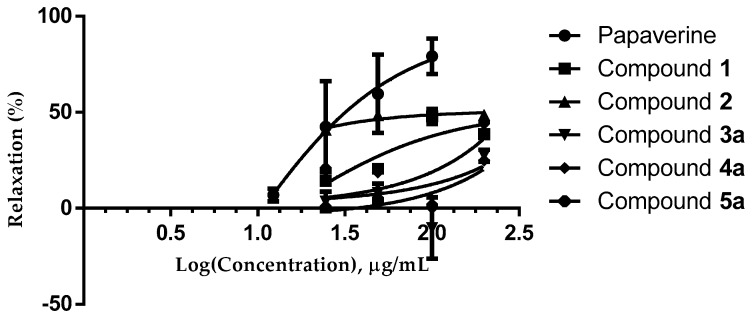
Inhibitory effect of compounds **1**, **2** and **3a**–**5a**, induced contractions in isolated guinea pig ileum, and compared with papaverine as a control (+).

The results presented in Figure 2 demonstrate that compounds **1** and **2** isolated from the heartwood of *H. campechianum* induced a concentration-dependent inhibition of the electrical stimulation-induced contractions in the guinea-pig ileum, as did the papaverine positive control.

Chromatographic separation allowed us to isolate five homoisoflavonoids-type compounds; two chalcones known as sapachalcone (**1**, EC_50_ = 16.06 ± 2.15) and 3-deoxysappachalcone (**2**, EC_50_ = 25.37 ± 3.47), that presented similar relaxing activity as papaverine (EC_50_ = 20.08 ± 2.0) and hematoxylol A tetraacetate (**3a**, EC_50_ = 204.5 ± 2.75), 4-*O*-methylhematoxylol tetracetate (**4a**, EC_50_ = 285 ± 11.0), and hematoxin diacetate (**5a**, EC_50_ = 203.1 ± 7.2). It must be noted that compounds **3a**, **4a** and **5a** had no significant relaxing activity (Table 1). These results suggest that the effect produced by the methanolic extract is mainly caused by the chalcones. With this finding, both the biological activity of *H. campechianum*, as well as its use in traditional medicine were demonstrated. However, further studies are required in order to determine the biological activity of this plant for specific diseases, in order to develop treatments focused on solving particular illness.

### 2.3. Structural Elucidation of Homoisoflavoids

#### 2.3.1. Sappanchalcone (**1**)

Chromatographic fractionation of HcF6 and HcF7 allowed for the purification of **1** which was obtained as a yellow powder with a melting point of 166.5 °C. The ^1^H-NMR spectra of **1** showed three systems; α,β unsaturated double bond system in δ 7.37 (d, 15.7 Hz, H-2α), and 7.49 (d, 15.7 Hz, H-3β); aromatic ABX system δ 7.11 (d, 2.2 Hz, H-2), 6.79 (d, 8 Hz, H-5) and 6.99 (dd, 2.2,8 Hz, H-6); another aromatic ABX system δ 6.52 (d, 2.2 Hz, H-3′), 6.46 (dd, 2.2, 8.4 Hz, H-5′) and 7.57 (d, 8.4 Hz, H-6′), and finally an oxigenated base signal at δ 3.9 (s, OCH_3_). The methoxyl position was defined unambiguously to be at C-2′ (δ 162.5) due to the long-range correlation (HMBC) between protons OCH_3_ (δ 3.9, s). On the basis of this information, the natural product was identified as sappanchalcone (**1**). Direct comparison of spectroscopic data (see Table 2 and Table 3) from this compound displayed a high similarity with those previously described for 2′-methoxy-3,4,4′-trihydroxychalcone or named sappanchalcone (**1**) [11].

#### 2.3.2. 3-Deoxysappachalcone (**2**)

Compound (**2**) was obtained as an orange powder with a melting point of 242 °C. The NMR spectra of ^1^H and ^13^C, showed the same signals as compound (**1**) (see Table 1 and Table 2), except for one of the ABX systems of aromatic rings, which is now AB in 7.49 (dd, 1.8, 8.4 Hz, H-2, H-6), and 6.81 (dd, 1.8, 8.4 Hz, H-3, H-5). Direct comparison of spectroscopic data with those described indicate that this compound corresponds to 2′-methoxy-4,4′-dihydroxychalcone, also known as 3-deoxysappanchalcone (**2**) [11].

**Figure 3:**
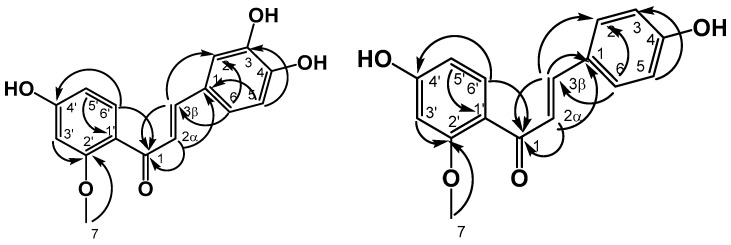
HMBC correlations in compounds **1** and **2**.

The carbon-hydrogen assignment was performed with HSQC and the correlations at two and three bonds were established using the HMBC spectra (Figure 3).

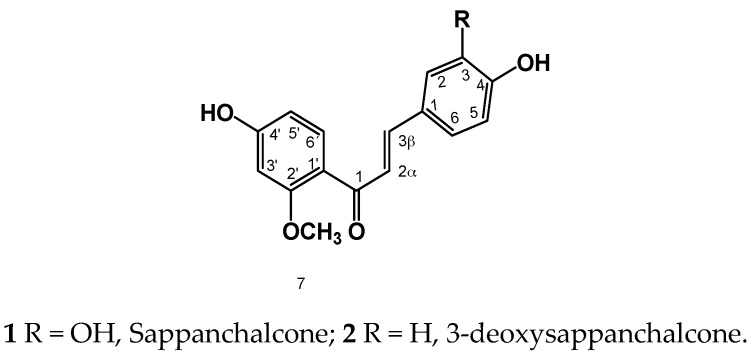



#### 2.3.3. Hematoxylol A Tetraacetate (**3a**)

Compound (**3**) was isolated as a white powder with a melting point of 239 °C. According to the analysis of NMR spectroscopic data (see Table 2 and Table 3) and the comparison with data described in the literature, this compound was identified as 3,4,10,11-tetrahydroxy-7,8-dihidroxy-6*H*-dibenz[*b*,*d*]oxocin-7-one (see Figure 1) on the basis of the DEPT, COSY HSQC, and HMBC spectra of its peracetate derivative (**3a**), also known as hematoxylol A(**3**), which had been previously isolated from *Haematoxylum campechianium* and tested as a protein tyrosine kinase inhibitor [6,13].

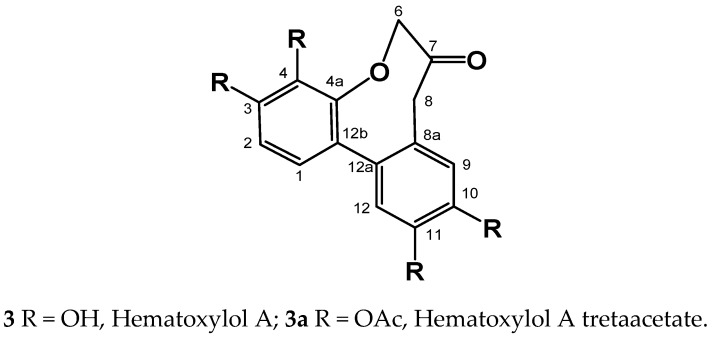



#### 2.3.4. 4-*O*-Methylhematoxylol Tetracetate (**4a**)

Compound (**4**) was isolated as a yellow powder with a melting point of 98.2 °C. On the basis of the DEPT, COSY HSQC, and HMBC spectra (see Table 1 and Table 2) of its peracetate derivative (**4a**) and the comparison with data described in the literature this compound was identified as 4-*O*-methylhematoxylol tetraacetate (**4a**). The natural compound (**4**) was isolated from *Haematoxylum campechianium* and tested as a protein tyrosine kinase inhibitor [6,13].

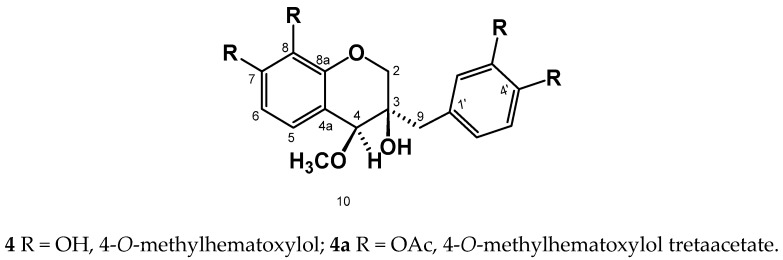



#### 2.3.5. Hematoxin Diacetate (**5a**)

Compound (**5**) was isolated as a yellow powder with a melting point of 136.9 °C. On the basis of the DEPT, COSY, HSQC, and HMBC spectra (see Table 1 and Table 2) of its peracetate derivative (**5a**), and the comparison with data described in the literature this compound was identified as hematoxin diacetate (**5a**). The natural compound (**5**) was isolated from *Haematoxylum campechianium* and tested as a protein tyrosine kinase inhibitor [6,13].

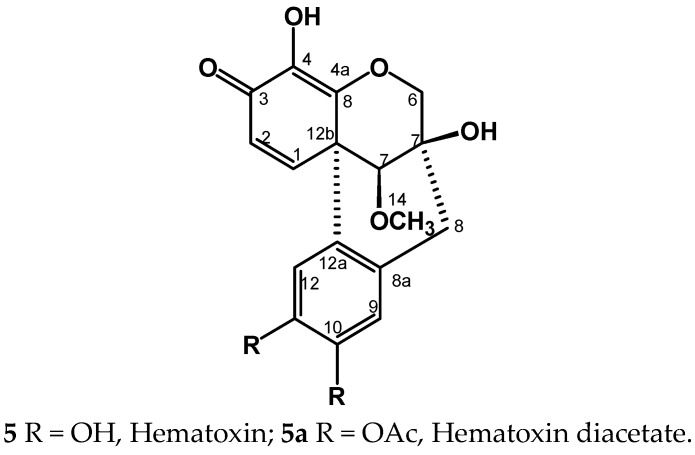



## 3. Materials and Methods

### 3.1. General Experimental Procedures

Melting points were obtained on a Termo Scientific IA9000 series melting point apparatus (Electrothermal, Essex, UK) and were left uncorrected. All NMR spectra were recorded on a Bruker Advance III HD-600 at 600 MHz for ^1^H-NMR, NOESY, ^1^H-^1^H COSY, HSQC, and HMBC, and 150 MHz for ^13^C-NMR and DEPT in CDCl_3_, CD_3_OD, CD_3_COCD_3_ and DMSO. Chemical displacements are reported in ppm relative to TMS.

HPLC analysis was performed using a Waters HPLC instrument equipped with Waters 996 UV photodiode array detector (900) set at 280 nm, and employing a packed column SUPELCOSIL LC-F^®^ 25 cm × 4.6 mm, 5 µm, at a flow rate of 0.9 mL/min, and a gradient system of TFA to 0.5%, and H_2_O (A:B) as the mobile phase with the following solvent ratios: A:B; 100:0 (0–1 min); 95:5 (2–3 min); 70:30 (4–20 min); 50:50 (21–23 min); 20:80 (24–25 min); 0:100 (26–27 min); and, 100:0 (28–30 min). Total running time of samples was 30 min, with a 10 μL injection. The detection wavelength was scanned at 190–400 nm.

For quantification of the isolated compounds, 5 mg in one milliliter of methanol were dissolved in dilution series of 25, 50, 100 and 200 g/mL.

### 3.2. Plant Material

The heartwoods of *Haematoxilumn campechanium* L. (7.5 kg), were collected in Cunduacán Tabasco, Mexico in February 2016. The voucher specimen of this material (No. 35455) was deposited in the herbarium of Juarez Autonomous University of Tabasco at Villahermosa, Tabasco, México.

### 3.3. Extraction and Chromatographic Fractionation of Heartwood

The dried and powdered heartwood was successively extracted three times with methanol (14 L, 3 times) for 24 h, at a room temperature. The obtained methanolic extract was evaporated to dryness with a rotary evaporator under reduced pressure (Heidolph G3, Schwabach, Germany), producing a residue of 300 g.

Methanolic extract (200 g) was adsorbed in silica gel and applied to a column of silica gel for gravity (1000 g, 70–230 mesh, Merck, Darmstadt, Germany). A gradient of dichloromethane/methanol was utilized to elute the column, collecting nine fractions (F1–F9) of 1 L each. The fractions were concentrated in a rotary evaporator under a reduced pressure of HcF1 (100:0, 1.5 g), HcF2 90:10, 3.8 g), HcF3 (80:20, 5.2 g), HcF4 (70:30, 3.5 g), HcF5 (60:40, 6 g), HcF6 (50:50, 13.57 g), HcF7 (40:60, 5 g), HcF8 (30:70, 17.67 g), Hc9 (0:100, 12 g). Fractions 5–9 were evaluated for the inhibition of electrically contractions in ileum isolated from guinea pig.

### 3.4. Isolated of the Chalcones (***1***,***2***) and Homoisoflavonoids Peracetate (***3a**–**5a***)

The fractions (HcF6, HcF7 and HcF8) were subjected to a chromatographic fractionation in column. HcF7(4.5 g) was adsorbed and applied to a column of silica gel (175 g, 70–230 mesh, Merck) and eluted with a gradient system with a dichloromethane/methanol with an increase in polarity of 5%, obtaining 35 fractions (HcF7-F1 to HcF7-F14) of 100 mL each. A successive of chromatographic columns in the normal phase of the most active fraction (HcF7-F7), and finally a reverse phase chromatographic column (10 g, RP-18, 40–63 µm, Merck) with a mobile phase water/acetonitrile in gradient system (samples of 10 mL), allowed the obtainment of the known chalcones 3′-deoxy-sappanchalcone (**2**, 48.3 mg) and sappanchalcone (**1**, 50.6 mg). They were identified by NMR (^1^H, ^13^C, COSY, HSQC, and HMBC).

The fraction HcF8(14 g) was separated using a chromatographic column (10 × 50 cm) with normal phase silica gel (170 g, 70–230 mesh, Merck) and eluted with a gradient system with dichloromethane/methanol with an increase in polarity of 5%, obtaining 105 fractions (HcF8-F1 to HcF8-F18) of 100 mL each. The 18 fractions were grouped according to their similarity in TLC.

HcF8-F5-6 (2.2 g) was purified under a reverse phase chromatographic column (20 g, RP-18, 40–63 µm, Merck) and eluted with a mobile phase water/acetonitrile in a gradient system (samples of 12 mL) with an increase of 2%, obtaining 18 fractions F4 (94:6, 251.3 mg), F5 (90:10, 153.7 mg), F6 (88:12, 683 mg). HcF8-F5-6-F6 was purified under a reverse phase chromatographic column (10 g, RP-18, 40–63 µm, Merck) with a mobile phase water/acetonitrile in a gradient system (samples of 12 mL) that allowed to obtain 12 fractions, among them F5 (88:12, 18.2 mg) and F6 (88:12, 32.2 mg). Fractions F5-F6 and HcF8-F5-6-F4 were chemically derivatized by means of an acetylation reaction. Both were separated using a chromatographic column with normal phase silica gel (10 g, 70–230 mesh, Merck) and eluted with a gradient system with dichloromethane/acetone with an increase in polarity of 2%, and collecting samples of 10 mL, giving the homoisoflavonoids known as 4-*O*-methylhematoxylol (**4a**, 13.2 mg) of the fraction HcF8-F5-6-F6-F5-6-F4, and the HcF8-F5-6-F6-F5-6-F8 a hematoxin (**5a**, 9.8 mg), and hematoxylol A (**3a**, 134 mg ) of the fraction HcF8-F5-6-F4-F1. They were identified by NMR (^1^H, ^13^C, COSY, HSQC, and HMBC). Compounds **1**, **2** and **3a**–**5a**, were evaluated for the inhibition of electric contractions in ileum isolated from guinea pig.

### 3.5. Reaction of Acetylation

Fractions HcF8-F5-6-F4 (251 mg) and HcF8-F5-6-F6-F5-6 (50.4 mg), were treated with Ac_2_O (3 mL) and pyridine (1 mL) for 2 h [19]. The reaction was stopped with ice (2 g), and ethyl acetate (50 mL) was added, forming two phases (AcOEt and water). Phase of AcOEt was concentrated in rotary evaporator to give of HcF8-F5-6-F6-F5-6 and HcF8-F5-6-F4. These fractions were purified by column chromatography to give compounds (**3a**, **4a** and **5a**). The structures of these compounds were identified on the basis of 1D and 2D NMR techniques, and by comparison with literature data [6,11,13]. See data as supplement Figures S1–S25.

### 3.6. Model of Ileum Isolated Guinea Pigs

To evaluate the spasmolytic activity, the experimental model of electrically induced guinea pig isolated ileum was performed. Guinea pigs of either sex (250–500 g) were used and subjected to cervical dislocation. Ethical guidelines for the handling and slaughter of experimental animals indicated by the American Veterinary Medical Association (AVMA, 2001) were followed.

(Official Mexican Standard NOM-062-ZOO-1999). Their abdomens were opened. Their ileum were removed and maintained in Petri dishes containing Tyrode’s solution, and constantly aerated with carbogen gas (O_2_ 95%, CO_2_ 5%). Portions of about 1.5 cm length of the tissue were mounted in a set of 3 mL chambers. One end of the tissue was attached to the bottom of the chamber to an electrode while the other was attached with a silk thread to a force transducer, which was connected to an acquisition system (PanLab, BIOPAC Systems, Goleta, CA, USA). After 30 min of an adaptation period, the tissue was electrically stimulated (25 V, 5 mS, 1 Hz, 5 S, every 2 min; with a Grass stimulator) by isolated tungsten electrodes connected to the end of the tissue. Induced contractions were recorded and after homogeneous response, different concentrations of drugs under study were added into the chamber, and the ability for inhibiting the electrically induced contraction was evaluated [20]. The positive control used was papaverine.

The Tyrode’s solution (Krebs-Henseleit) was prepared as follows in mM: NaCl (137), C_6_H_12_O_6_ (**5**), NaHCO_3_ (11.9), CaCl_2_·2H_2_O (2.7), KCl (5.4), MgCl_2_·6H_2_O (0.5), y NaH_2_PO_4_·H_2_O (0.45) and diluted to a volume of 2 L with distilled water. The pH was adjusted to 7.4.

### 3.7. Preparation of Extracts, Fractions and Compounds for Evaluations

The methanolic extract, fractions, and compounds were diluted in Tyrode’s solution and PVP (polyvinylpyrrilodine, 1:2) for the less polar.

### 3.8. Statistical Analysis

The effect in this model was expressed as the mean ± standard deviation (SD). From *n* = 3 independent experiments were performed in triplicates, and were determined by linear regression analysis using GraphPad Prism 6.0 Software(GraphPad Software, San Diego, CA, USA). The values of the mean effective concentrations (EC_50_) and maximum effects (E_max_) were obtained from the concentration-response curves. The data were analyzed by a one-way ANOVA and the Tukey test. Differences between the means were considered to be significant when *p* ˂ 0.05.

## 4. Conclusions

Five compounds of *H. campechianum* methanolic extract were isolated and characterized: two chalcones known as sappanchalcone (**1**), 3′-deoxy-sappanchalcone (**2**), three homoisoflavonoids derivatized known as hematoxylol A (**3a**), 4-*O*-methylhematoxylol (**4a**), and hematoxin (**5a**). The correct structures were assigned by detailed spectroscopy analysis of NMR in one and two dimensions. The most bioactive chalcones **1** and **2** were isolated from the spasmolytic fraction (Hc-F7). In the biological test in vitro, extract, fractions, and compounds **1** and **2**. These results validate the traditional use of this plant.

## Figures and Tables

**Table 1:** Inhibitory effect of the methanolic extract, fractions (HcF5 to HcF9) and compounds (**1**–**2**, **3a**–**5a**) of *Haematoxilum campechianum* induced contractions in isolated guinea pig ileum (Effective concentration fifty EC_50_, relaxation E_max_).

Extract, Fraction and Compounds	Effective Concentration Fifty (CE_50_ = µg/mL)	Relaxation (%) Emax
Extract MeOH	62.11 ± 3.23 ^b^	85.16 ± 6.34 ^d^
Fraction		
HcF5	346.7± 19.32 ^f^	56.7 ± 1.8 ^c^
HcF6	324.9 ± 10.15 ^f^	80.9 ± 2.5 ^d^
HcF7	61.75 ± 3.55 ^b^	84.6 ± 1.7 ^d^
HcF8	343.7 ± 10.04 ^f^	95.1 ± 1.2 ^e^
HcF9	125.5 ± 10.65 ^c^	24.45 ±1.6 ^a^
Compound		
**1**	16.06 ± 2.15 ^a^	50.9 ± 2.2 ^b,c^
**2**	25.37 ± 3.47 ^a^	48.5 ± 2.0 ^b^
**3a**	204.5 ± 2.75 ^d^	85.78 ± 2.7 ^d^
**4a**	285 ± 11.0 ^e^	ND
**5a**	203.1 ±7.2 ^d^	100 ±0.3 ^e^
Papaverine (+)	20.08 ± 2.0 ^a^	85.72 ± 2.8 ^d^

* Values are mean ± SD., *n* = 3 independent experiments performed in triplicates, and were determined by linear regression analysis using GraphPad Prism 6.0 Software; ^a–f^ Values are statistically significant at *p* < 0.05. ND = not determined.

**Table 2:** ^13^C-NMR data of homoisoflavonoids **1**–**5** (**1** and **2** in CD_3_OD, **3a**–**5a** in CDCl_3_, 150 MHz).

Position	1	2	3a	4a	5a
2α	124.8	125.2			
3β	144.2	144.2			
1 C=O	192.5	193.2			
1′	121.8	121.8		134.3	
2′	162.5	162.6		125.7	
3′	99.8	100.3		141.6	
4′	164.4	164.6		140.8	
5′	108.7	109.0		122.9	
6′	133.4	134.1		129.0	
1	128.7	128.2	126.8		144.1
2	114.9	131.5	120.6	69.4	130.1
3	146.8	117.0	136.5	69.9	180.4
4	149.6	161.3	132.7	76.2	136.6
4a			149.2	118.2	160.7
5	116.2	117.0		128.5	
6	123.0	131.5	76.9	113.9	77.5
7	55.87	56.28	203.6	143.6	70.0
8			49.0	130.9	38.67
8a			142.0	146.6	133.9
9			125.2	38.2	123.3
10			142.1	56.2	141.8
11			144.3	167.7	140.6
12			125.0	168.2	121.7
12a			136.3		130.0
12b			131.0		52.7
13			167.9	168.4	83.75
14			168.5	168.4	62.51
15			168.6	20.64	168.0
16			168.7	20.65	168.0
17			20.80	20.3	20.52
18			21.23	20.3	20.68
19			21.16		
20			21.16		

**Table 3:** ^1^H-NMR data of homoisoflavonoids **1**–**5** (**1** and **2** in CD_3_OD, **3a**–**5a** in CDCl_3_, 600 MHz, *J* in Hz).

Position	1	2	3a	4a	5a
2α	7.37, d, 15.7	7.40, d, 15.7			
3β	7.49, d, 15.7	7.55, d, 16.5			
2′				7.18, s	
3′	6.52, d, 2.2	6.51,d, 2.2			
5′	6.46, dd, 2.2, 8.4	6.45, dd, 2.2, 8.4		7.14, d, 8.07	
6′	7.57, d, 8.4	7.57, d, 8.4		7.21, d, 8.07	
1			7.28, d, 8.4		7.08, d, 9.9
2α	7.11, d, 2.2	7.49, dd, 1.8, 8.4	7.17, d, 8.8	3.94, d, 11.3	6.63, d, 10.2
2β				4.19, d, 11.0	
3		6.81, dd, 1.8, 8.4			
4				3.7, s	
5	6.79, d, 8.0	6.81, dd, 1.8, 8.4		7.02, d, 8.07	
6α	6.99, dd, 2.2, 8.0	7.49, dd, 1.8, 8.4	4.5, s	6.72, d, 8.07	3.86, dd, 2.2, 11
6β					4.21, d, 11
7	3.9, s	3.88, s			
8α			3.6, s		3.10, d, 16.5
8β					3.40, d, 16.1
9α			7.2, s	2.84, d, 13.5	7.03, s
9β				3.02, d, 14.3	
10				3.34	
12			7.18, s		6.78, s
13					3.6, s
14					3.6, s
15				2.3, s	
16				2.3, s	
17			2.35, s	2.3, s	2.24, s
18			2.33, s	2.3, s	2.26, s
19			2.3, s		
20			2.3, s		

## Supplementary Materials

The following are available online: Figures S1–S25.
