# Supplementary material for: Homoisoflavonoids and Chalcones Isolated from Haematoxylum campechianum L., with Spasmolytic Activity
Source: Molecules. 2017 Aug 24;22(9):1405. doi: 10.3390/molecules22091405 (PMC6151460; doi:10.3390/molecules22091405)
Supplement: Supplementary file 1 [file molecules-22-01405-s001.pdf]

Homoisoflavonoids and chalcones isolated from *Haematoxylum campechianum* L., with spasmolytic activity.

Armando Escobar-Ramos[1], Carlos Ernesto Lobato-García[1,*], Alejandro Zamilpa[2], Abraham Gómez-Rivera[1], Jaime Tortoriello[2],. Manasés González-Cortazar[2,*]

[1] University Juárez Autonomous of Tabasco, highway Cunduacán-Jalpa Km. 0.5, Cunduacán Tabasco, 86690, México; armando.escobar@ujat.mx; abgori@gmail.com; carloslobatogarcia@gmail.com

[2] Southern Biomedical Research, Mexican. Institute of Social Security, Argentina No. 1, Col. Centro, 62790 Xochitepec, Morelos, México; gmanases2000@gmail.com; azamilpa_2000@yahoo.com.mx; jtortora2@yahoo.es

* Correspondence: gmanases2000@gmail.com ; carloslobatogarcia@gmail.com; Tel.: + 52 (777) 3 612155

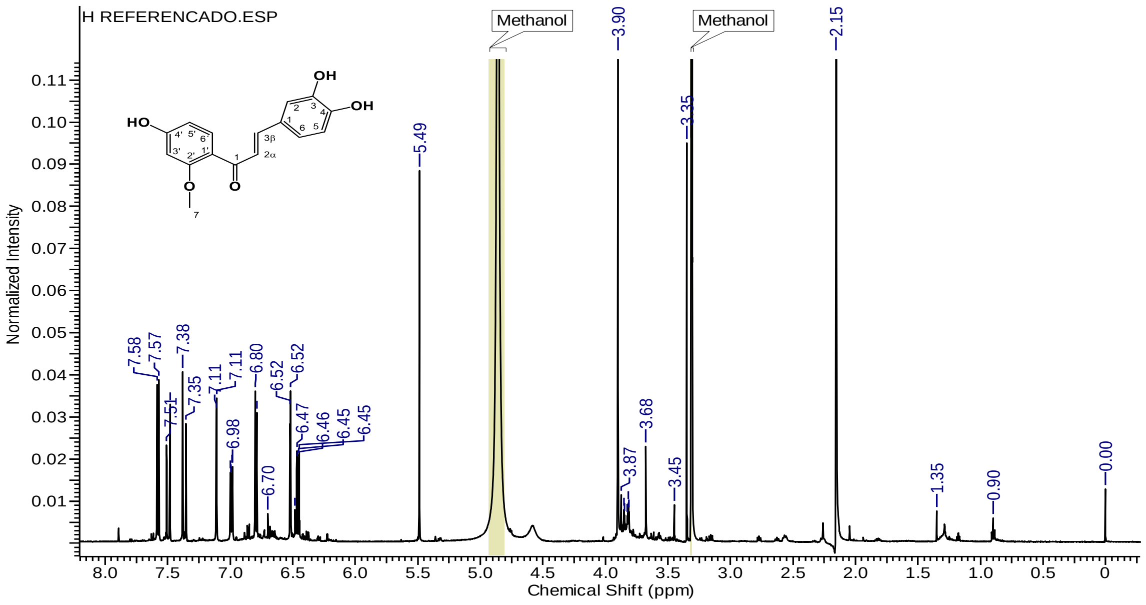

Fig. 1S ^{1}H NMR (CD_3OH, 600 MHz) Compound (**1**).

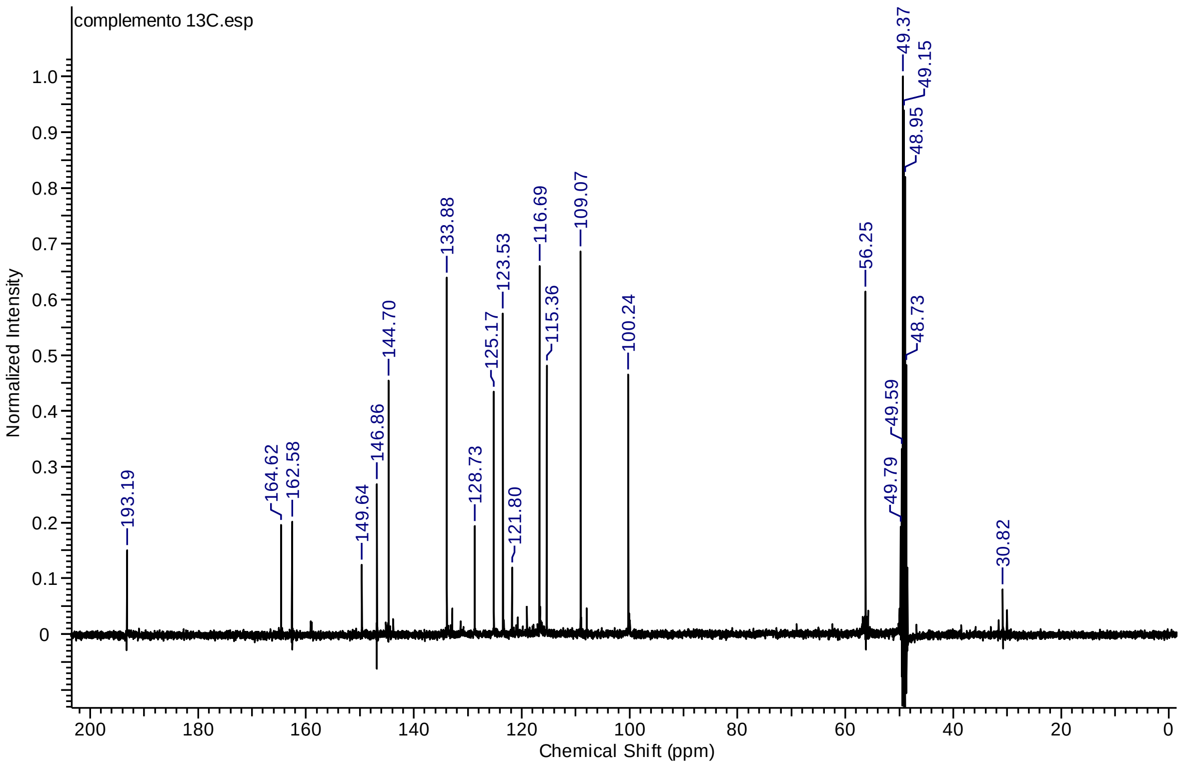

Fig. 2S ^{13}C NMR (CD_3OH, 600 MHz) Compound (**1**).

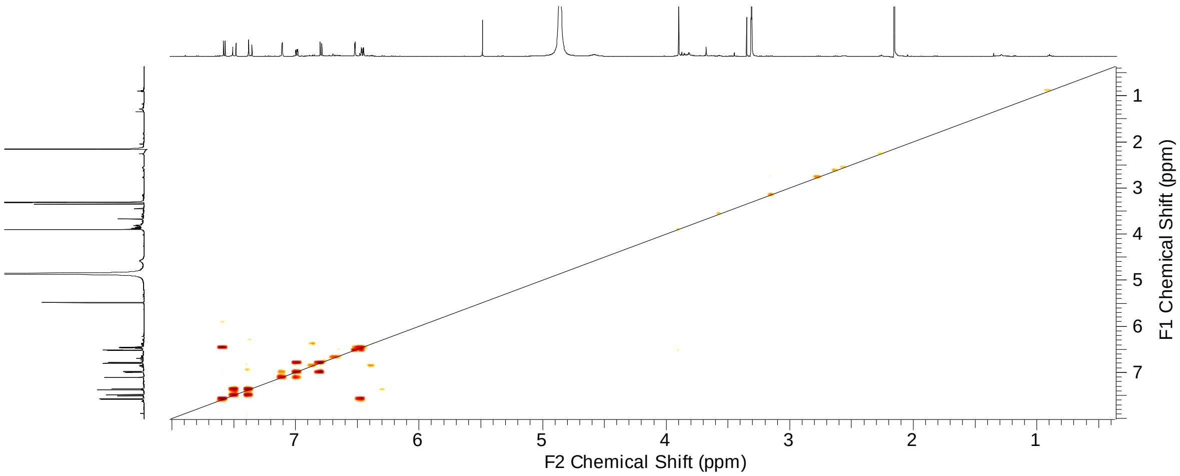

Fig. 3S COSY NMR (CD_3OH, 600 MHz) Compound (**1**).

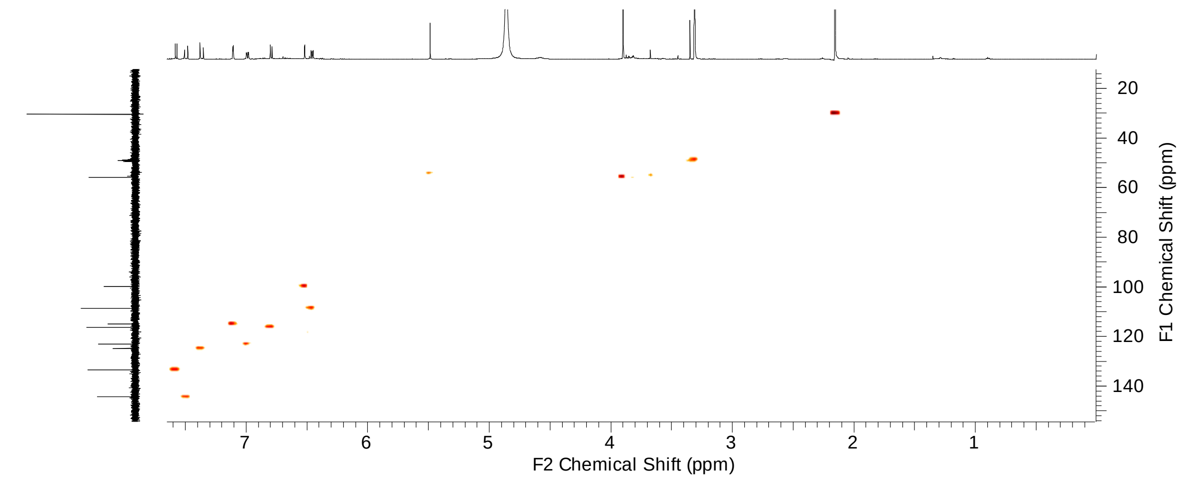

Fig. 4S HSQC (CD_3OH, 600 MHz) Compound (**1**).

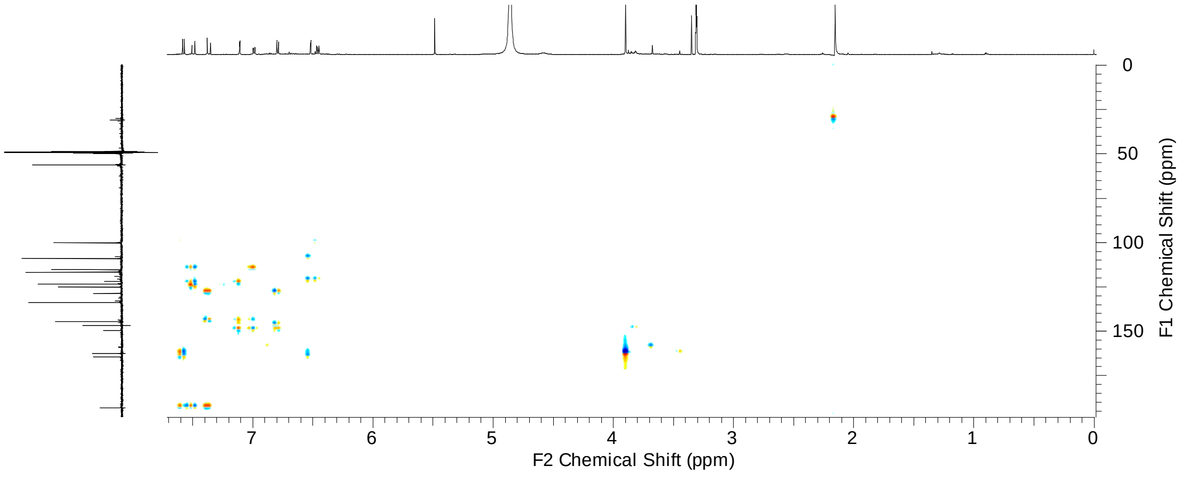

Fig. 5S HMBC (CD_3OH, 600 MHz) Compound (**1**).

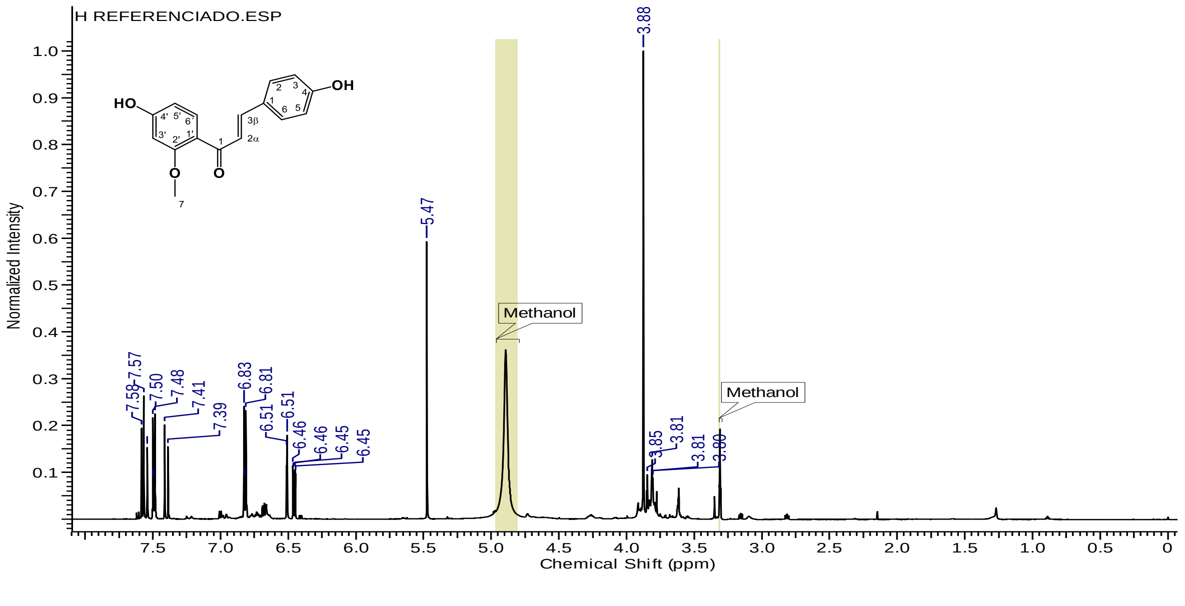

Fig. 6S ^{1}H NMR (CD_3OH, 600 MHz) Compound (**2**).

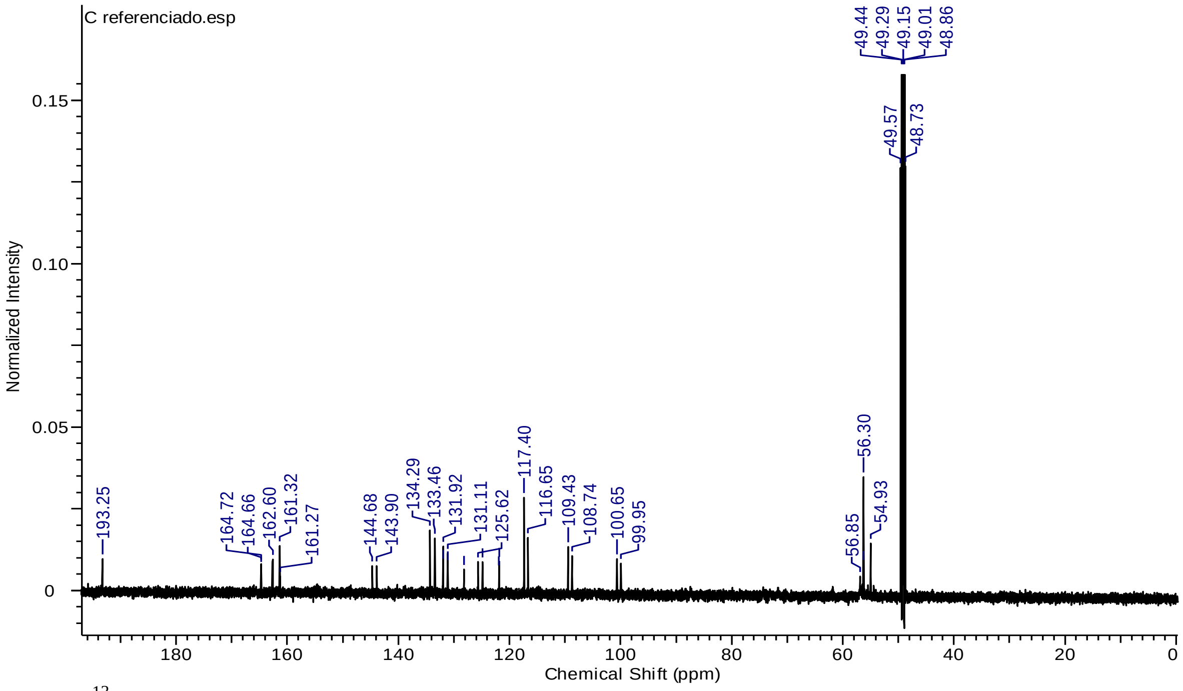

Fig. 7S ^{13}C NMR (CD_3OH, 600 MHz) Compound (**2**).

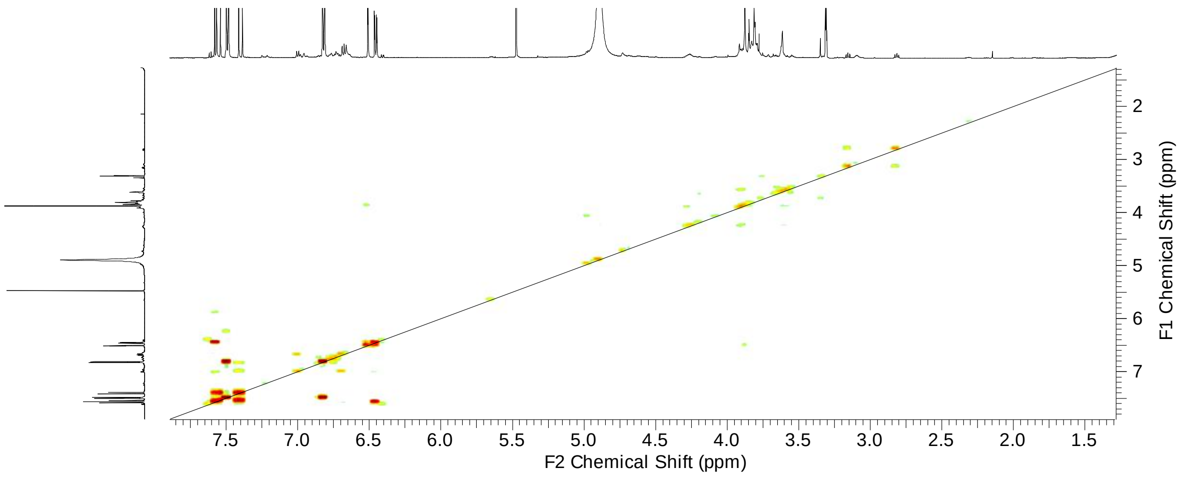

Fig. 8S COSY NMR (CD_3OH, 600 MHz) Compound (**2**).

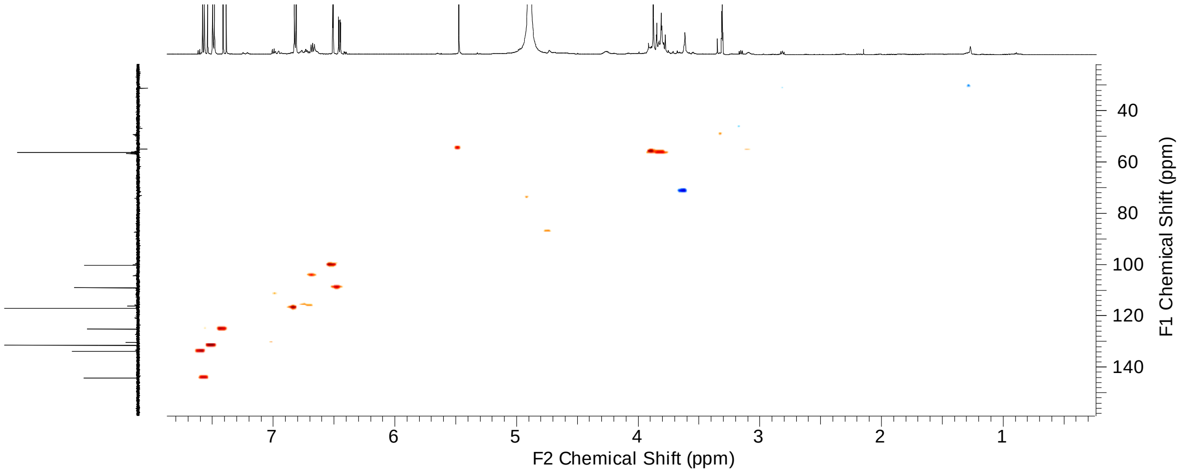

Fig. 9S HSQC NMR (CD_3OH, 600 MHz) Compound (**2**).

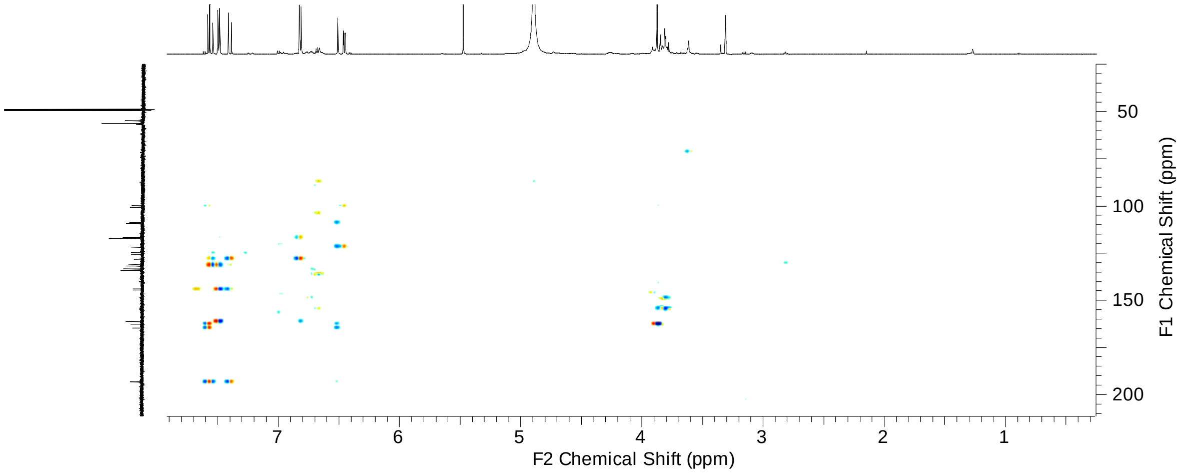

Fig. 10S HMBC NMR (CD_3OH, 600 MHz) Compound (**2**).

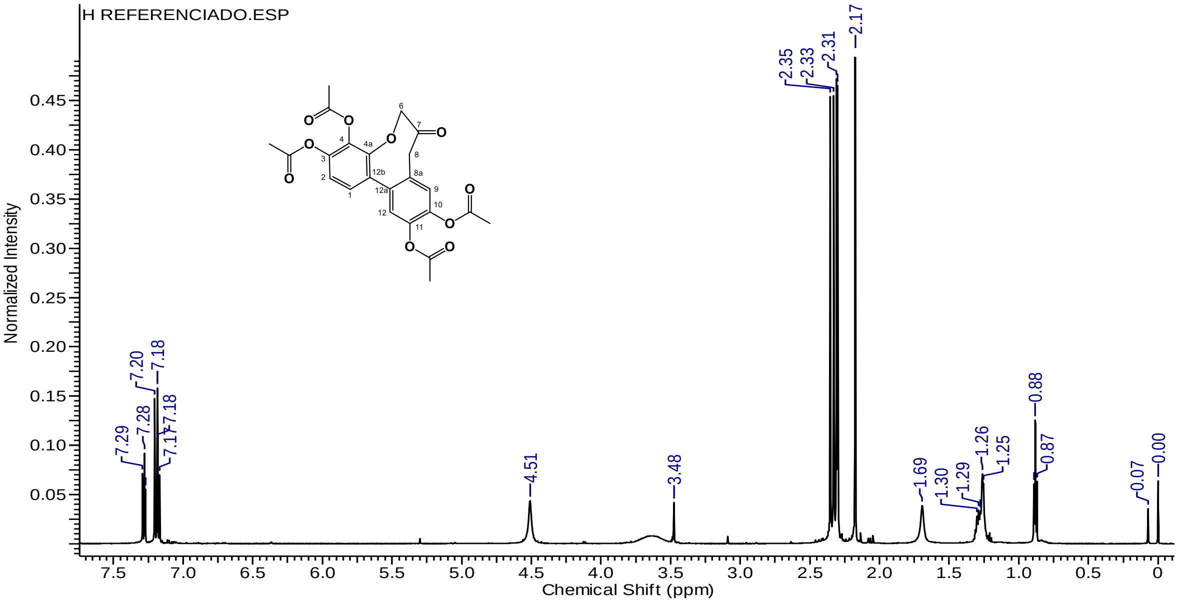

Fig. 11S ^{1}H NMR ($CDCl_3$, 600 MHz) Compound (**3a**).

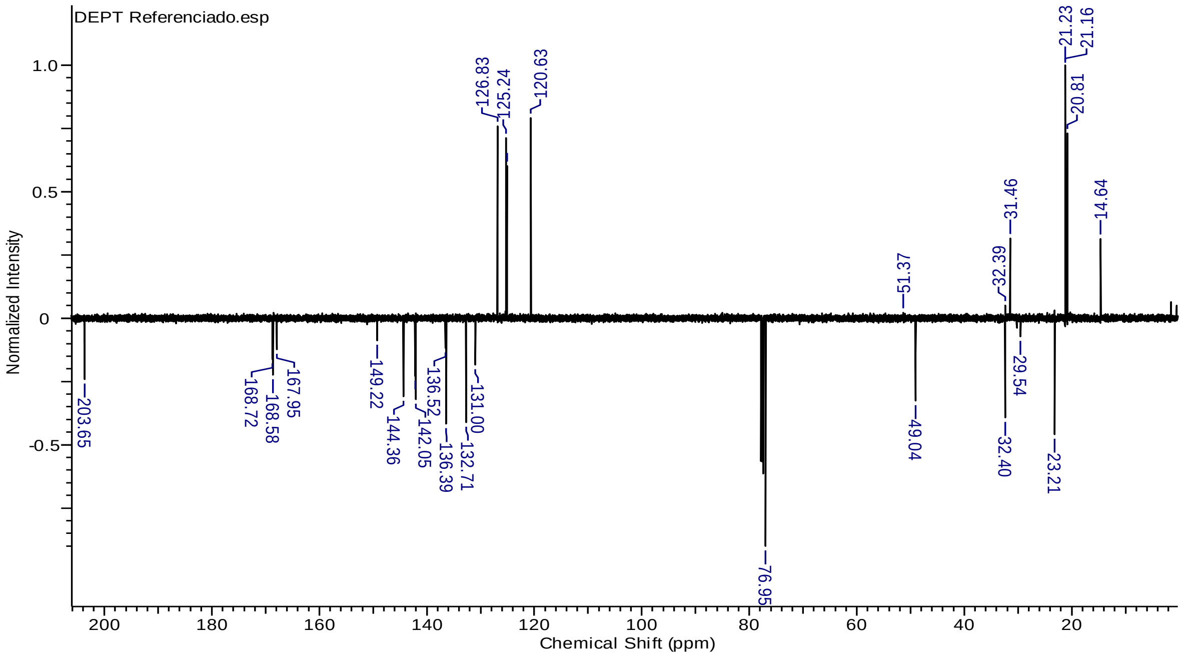

Fig. 12S DEPT NMR ($CDCl_3$, 600 MHz) Compound (**3a**).

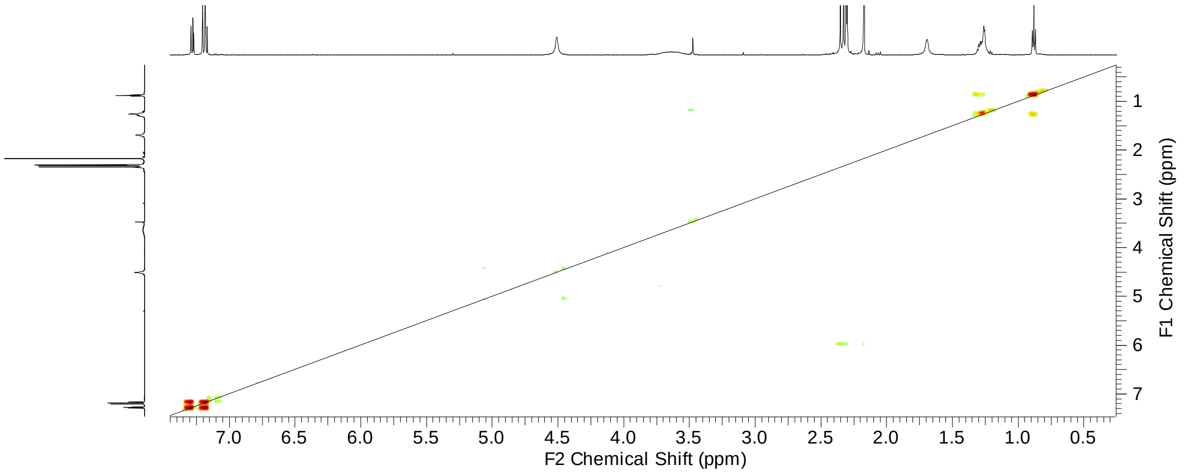

Fig. 13S COSY NMR ($CDCl_3$, 600 MHz) Compound (**3a**).

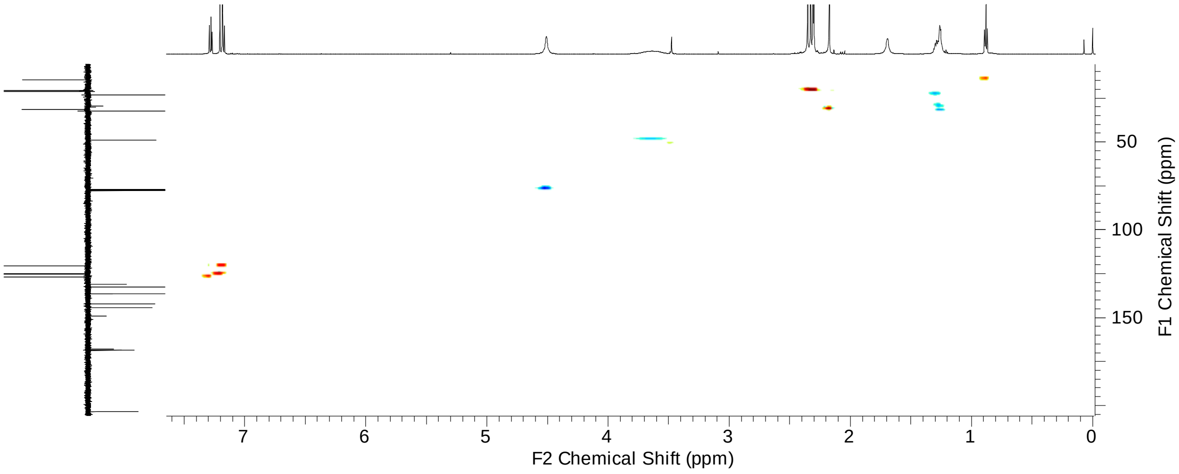

Fig. 14S HSQC NMR ($CDCl_3$, 600 MHz) Compound (**3a**).

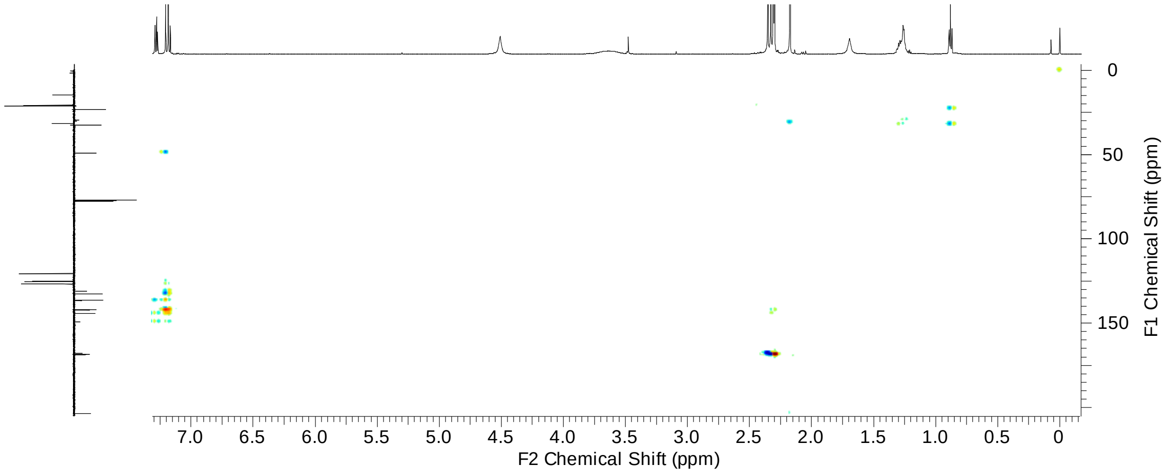

Fig. 15S HMBC NMR ($CDCl_3$, 600 MHz) Compound (**3a**).

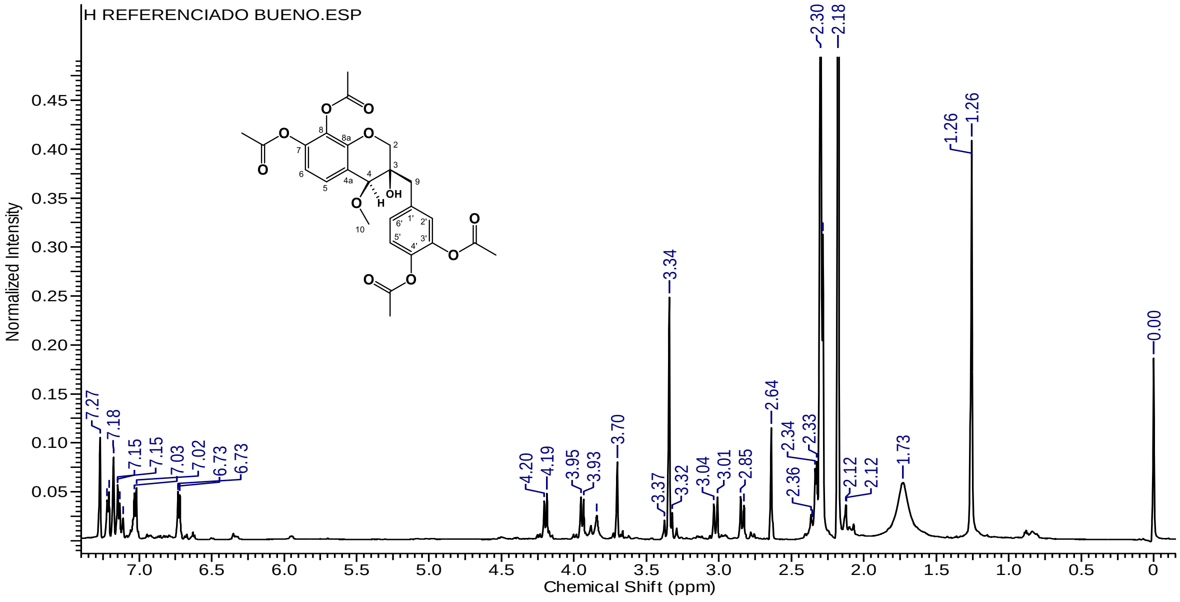

Fig. 16S ^{1}H NMR ($CDCl_3$, 600 MHz) Compound (**4a**).

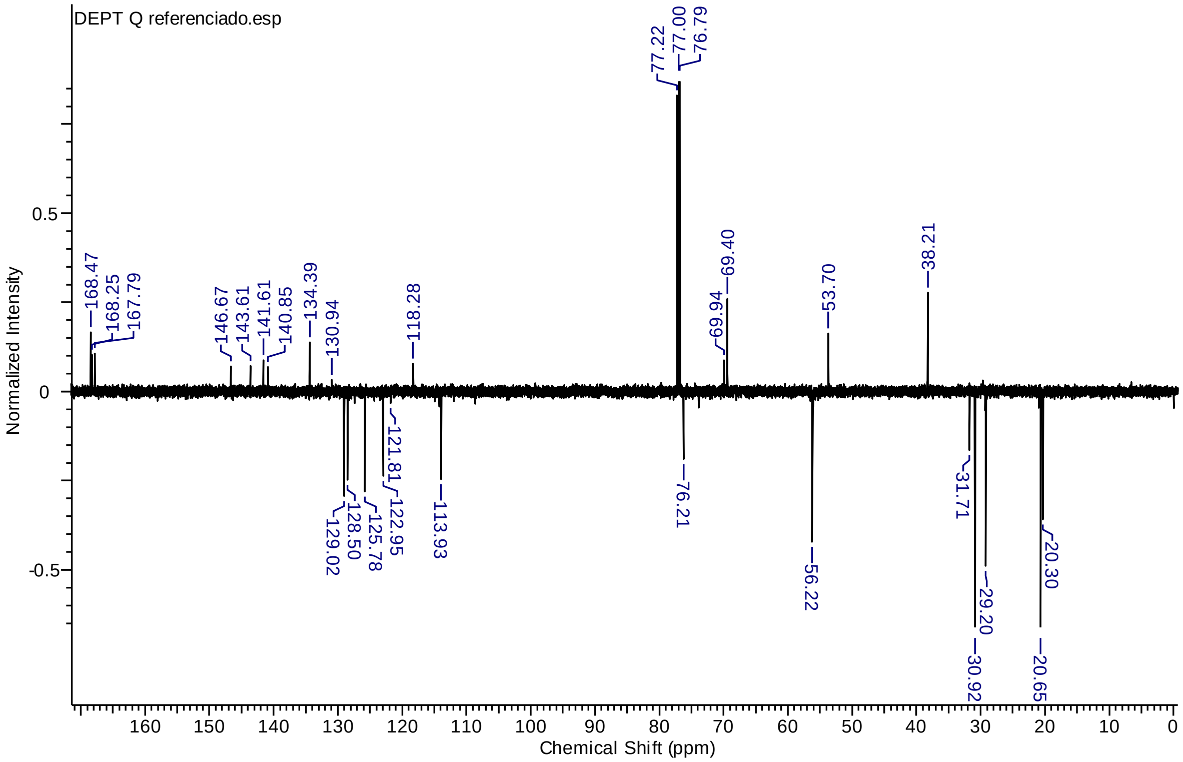

Fig. 17S DEPT NMR ($CDCl_3$, 600 MHz) Compound (**4a**).

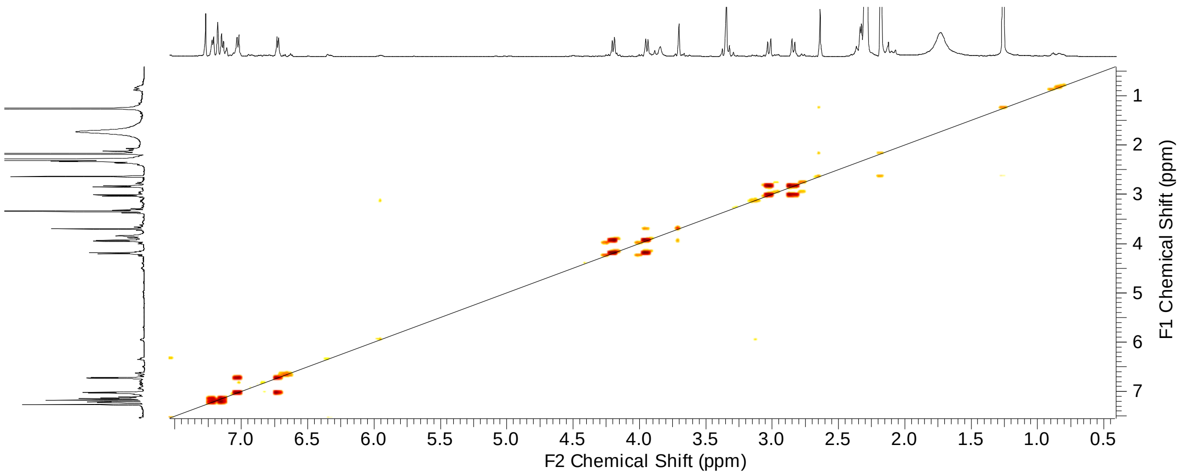

Fig. 18S COSY NMR ($CDCl_3$, 600 MHz) Compound (**4a**).

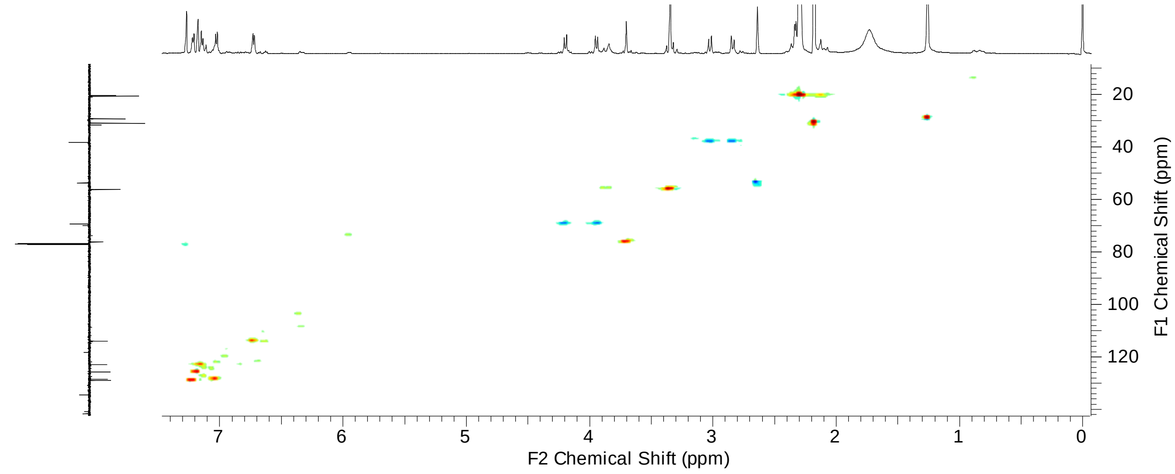

Fig. 19S HSQC NMR ($CDCl_3$, 600 MHz) Compound (**4a**).

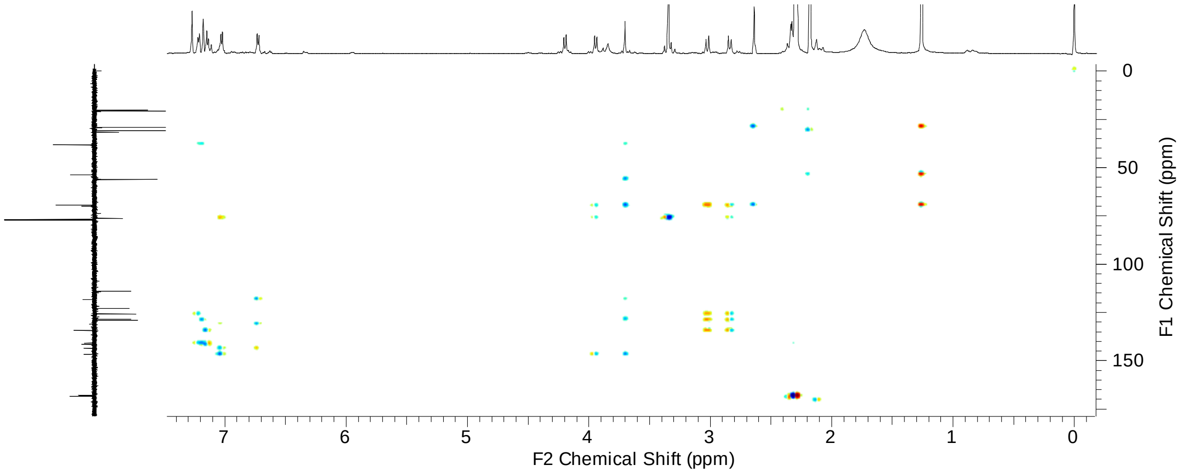

Fig. 20S HMBC NMR ($CDCl_3$, 600 MHz) Compound (**4a**).

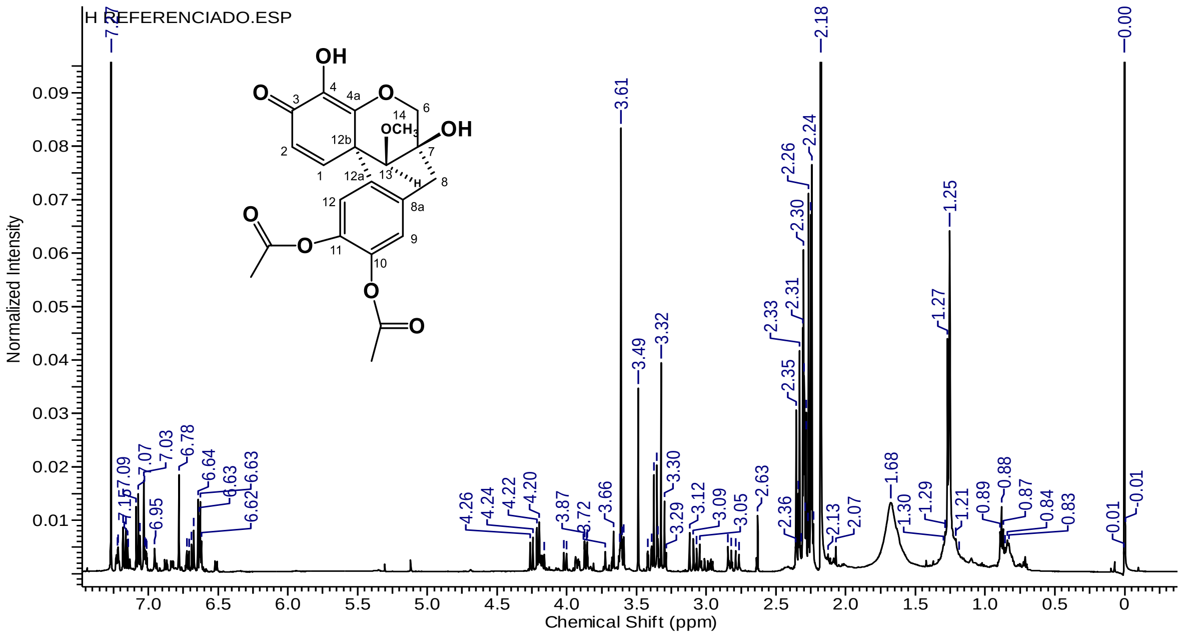

Fig. 21S ^{1}H NMR ($CDCl_3$, 600 MHz) Compound (**5a**).

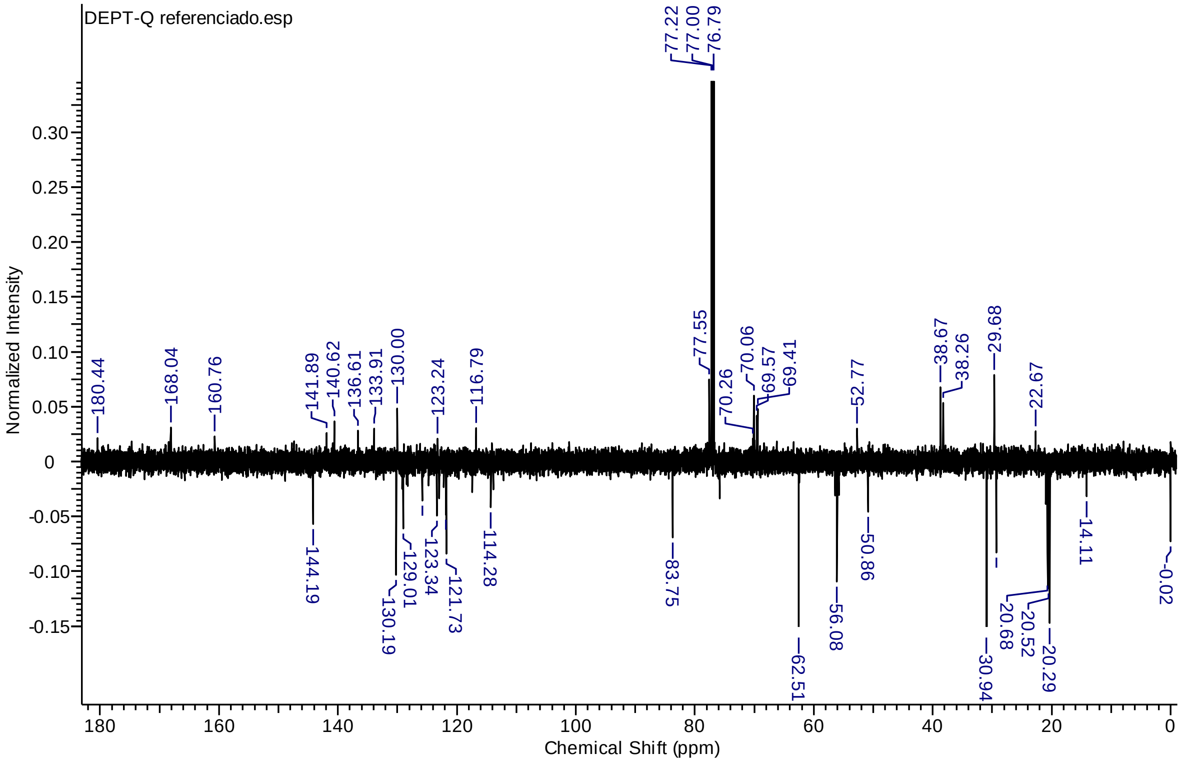

Fig. 22S DEPT NMR ($CDCl_3$, 600 MHz) Compound (**5a**).

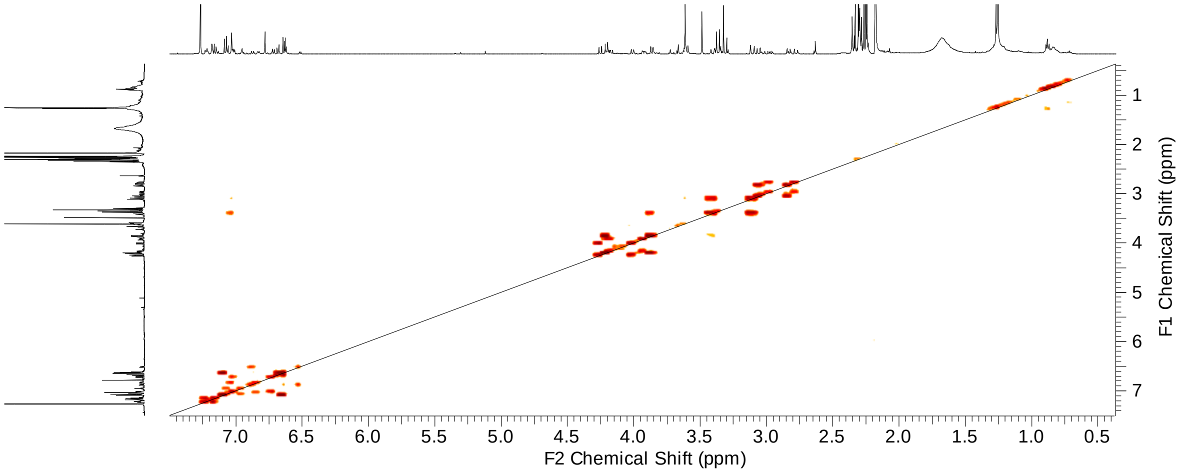

Fig. 23S COSY NMR ($CDCl_3$, 600 MHz) Compound (**5a**).

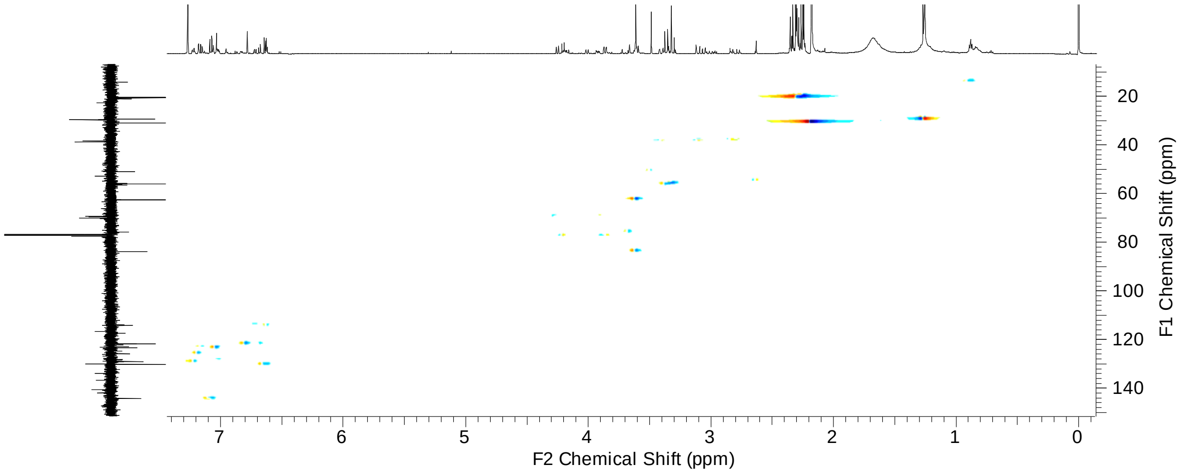

Fig. 24S HSQC NMR ($CDCl_3$, 600 MHz) Compound (**5a**).

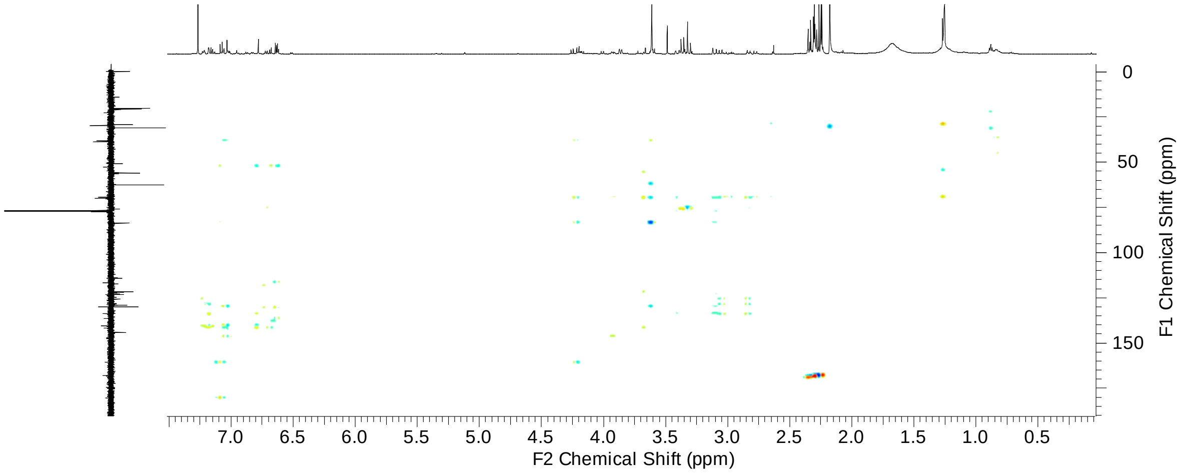

Fig. 25S HMBC NMR ($CDCl_3$, 600 MHz) Compound (**5a**).
